# The Assessment of Quality of the Root Canal Filling and the Number of Visits Needed for Completing Primary Root Canal Treatment by Operators with Different Experience

**DOI:** 10.3390/bioengineering9090468

**Published:** 2022-09-13

**Authors:** Krystyna Pietrzycka, Mateusz Radwanski, Louis Hardan, Rim Bourgi, Davide Mancino, Youssef Haikel, Monika Lukomska-Szymanska

**Affiliations:** 1 Department of Endodontics, Medical University of Lodz, 251 Pomorska Str., 92-213 Lodz, Poland; 2 Department of Restorative Dentistry, School of Dentistry, Saint-Joseph University, Beirut 1107 2180, Lebanon; 3 Department of Biomaterials and Bioengineering, INSERM UMR_S 1121, Biomaterials and Bioengineering, 67000 Strasbourg, France; 4 Department of Endodontics, Faculty of Dental Medicine, Strasbourg University, 67000 Strasbourg, France; 5 Pôle de Médecine et Chirurgie Bucco-Dentaire, Hôpital Civil, Hôpitaux Universitaire de Strasbourg, 67000 Strasbourg, France; 6 Department of General Dentistry, Medical University of Lodz, 251 Pomorska Str., 92-213 Lodz, Poland

**Keywords:** endodontists, general practicing dentists, root canal treatment, quality, undergraduate students

## Abstract

The main goal of root canal treatment (RCT) is to eradicate or essentially diminish the microbial population within the root canal system and to prevent reinfection by a proper chemo-mechanical preparation and hermetic final obturation of the root canal space. The aim of this study was to assess the quality of the root canal filling and the number of visits needed for completing RCT by operators with different experience, including dentistry students (4th and 5th year), general dental practitioners (GDPs), and endodontists. Data from medical records of 798 patients were analyzed, obtaining 900 teeth and 1773 obturated canals according to the inclusion and exclusion criteria. A similar number of teeth was assessed in each group in terms of density and length of root canal filling and number of visits. The larger number of visits and the lower quality of treatment was observed for 4th year students than for other groups (*p* < 0.05); in contrast, the endodontists needed the lowest number of visits to complete RCT and more often overfilled teeth than other operator groups (*p* < 0.05). Interestingly, no statistical difference in quality of root canal filling was noted between 5th year students, GPDs and endodontists. The treatment of lower teeth demanded statistically more visits than that of upper teeth (*p* < 0.05). The results of the study emphasize that most of the root canal filling performed by operators was considered adequate, regardless of tooth type, files used and number of visits.

## 1. Introduction

The purpose of root canal treatment (RCT) is to maintain the function of a tooth, cure disorders of the pulp, prevent and treat the diseases of periapical tissue. Apical periodontitis is mainly caused by the colonization of microorganisms due to dental caries, dental trauma, or iatrogenic exposure of the pulp tissue to various oral microbiota [1]. Therefore, the main goal of RCT is to eradicate or essentially diminish the microbial population within the root canal system and to prevent reinfection by a proper chemo-mechanical preparation and hermetic final obturation of the root canal space [2,3].

The results of endodontic treatment are evaluated with the use of clinical and radiological examination [4]. The clinical findings should define whether signs and symptoms of infection are present. The radiological examination allows assessing the quality of filling of the canal system and periapical tissue.

The success of RCT amounts up to 68–95% [5,6,7,8]. According to Schilder [2], it depends not only on the cleaning of the canal and its cone-shaped preparation but also on the proper filling of the entire canal system. The standards of RCT were described in the recommendations for endodontic treatment: consensus report of the European Society of Endodontology (ESE) [9]. On the other hand, the scope of knowledge and skills that an European dentist should demonstrate upon graduation was published by De Moor in the Undergraduate Curriculum Guidelines [10]. According to both documents, the correct filling of the canal should be homogeneous, without any voids within the canal filling (internal voids), but also between the filling and the walls of the root canal (external voids). Moreover, the root canal filling should end at the length of 0.5 to 2.0 mm from the apex of the tooth root [9]. On the postoperative radiograph, the light of the root canal between the end of the filling and the radiological apex should not be visible [9].

There are different models of teaching across the globe. Endodontics at the Medical University of Lodz is taught in the third (6th semester—15 h of theory and 30 h of practical training), fourth (7th and 8th semester—34 h of theory and 96h of practical training) and fifth year (9th semester—24 h of theory and 49 h of practical training) of the five-year course of dentistry. In clinical classes (4th and 5th year), the assistant—student ratio amounts up to 1:6. In German-speaking countries, endodontic education at dental schools is differentiated. Theory classes range from 1 to 70 h (15 h mean), and practical classes range from 3 to 78 h (39 h mean) [11]. The staff–student ratio varies between 1:4 and 1:38 (mean—1:15). In the UK and Spain, students spend 20 h on preclinical training and 50 h on clinical training [12,13]. In Spanish dental schools, the staff–student ratio during preclinical endodontic training ranges from 1:6 to 1:20 and from 1:6 to 1:10 during clinical practice [12]. In UK dental schools, the staff–student ratio in preclinical training ranges from 1:5 to 1:20, and supervising staff mainly consists of general dental practitioners (GDPs) with/without a special interest and training in endodontics. During clinical training, the ratio is from 1:4 to 1:6, and students are supervised by GDPs with a special interest and training in endodontics and endodontists [13].

According to the ESE, postgraduate specialty training programs in endodontology within Europe should last 3 years [14]. In Poland, endodontics along with conservative dentistry is recognized as a specialty after 3 years of training. In Spain, endodontics is not recognized as a dental specialty, and the postgraduate program in endodontics lasts 2 or 3 years [12]. The duration of the full-time course in endodontics takes usually 2 (University of Glasgow, University of Birmingham) or 3 years (University of London, University of Plymouth, The University of Manchester, King’s College London, Queen Mary University of London) in the UK. The average duration of the Advanced Dental Education Program in Endodontics in USA lasts two (University of Illinois, University of Pennsylvania) or three years (Indiana University School of Dentistry, New York University). The three-year program of endodontics takes place in The University of Hong Kong, British Columbia (Canada), Queensland in Australia and Amrita University Coimbatore in India.

The quality of primary RCT may differ among dentistry students, GDPs, and endodontists. These discrepancies are associated with different levels of knowledge, experiences, and dexterity. To our best knowledge, there is no study comparing the quality of primary RCT performed by operators with differentiated experience in Poland.

The aim of this study was to compare the quality of the final filling and number of visits needed for completing primary root treatment performed by operators with different experience. The null hypothesis is that there are no differences in the quality of the filling and number of visits after treatment in evaluated groups.

## 2. Materials and Methods

### 2.1. Study Group

The study was approved by the Bioethics Committee of the Medical University of Lodz (RNN/04/18/KE). All patient’s data remain confidential and have been used for research purposes only. Information on the performed treatment was introduced to a clinical patient card entered an electronic database with a restricted access code. Before the analysis, the data were anonymized. Patients were admitted from October 2017 to February 2019. RCT was carried out with the patients’ informed consent to participate in the study.

Sample size estimation revealed 377 patients needed for the survey. Calculations were completed with a margin of error of 5% and confidence level of 95%. The inclusion criteria consisted of single- or multi-rooted teeth demanding primary root treatment. Teeth with complex anatomy, roots with severe apical resorption, external or internal resorption, open apex and calcifications were excluded from the study. X-rays of low quality or with additional artifacts were not included in the study. According to inclusion and exclusion criteria, the final sample group included data from medical records of 798 patients, including 900 teeth and 1773 obturated canals.

All treated patients were admitted by dentistry students (Medical University of Lodz), GPDs at the Endodontics Department, and by endodontists at the Endodontics Clinic of Clinical Hospital in Lodz. Both institutions have the same location and equipment in terms of the dental materials used during endodontic treatment.

### 2.2. Root Canal Treatment Protocol

Only primary RCT was included in the study. All treatment procedures were carried out following the standards of the ESE [9]. After the examination, and preoperative X-ray in two angulations was made to confirm diagnosis. Next, RCT protocol was initiated. All procedures were performed under local anesthesia and isolation with the use of a rubber dam (Rubber-Dam, size medium, Cerkamed, Stalowa Wola, Polska). The choice of anesthetic depended on the patient’s health condition; articaine with a vasoconstrictor (Ubistesin 4%, Molteni, 3M, St. Paul, MN, USA) was used for healthy individuals, and when this anesthetic was contraindicated, mepivacaine was applied (Mepivastesin 3% Molteni, 3M, St. Paul, MN, USA). When trepanation of pulp cavity was accomplished, the chamber was prepared, and the canals were found. Loupes (2.5×) and microscope (8×) were applied when orifices could not be localized. The canal orifices were prepared to assure a straight line access to the canals; then, the working length (WL) was determined with sodium hypochlorite (NaOCl) in root canal and a C-PILOT file (VDW, Munich, Germany) using a Raypex 5 apex locator (VDW, Munich, Germany).

All root canals were shaped by the students with the step-back technique using RT files (Mani, Tochigi, Japan), and the Master Apical File (MAF) was #30–35 for all canals (determined after evaluation of the initial size of the physiological foramen). GDPs and endodontists used nickel–titanium (NiTi) rotary files. Depending on the anatomical characteristics of the teeth and clinicians’ preferences rotary NiTi files: ProTaper Next (Dentsply Maillefer, Ballaigues, Switzerland), Mtwo (VDW, Munich, Germany), E3 Azure (Poldent, Warszawa, Poland) and DC-taper 2H (SS White, Lakewood, NJ, USA) according to the manufacturer’s instructions were used. The canals were shaped with the X-smart Endodontic Motor (Dentsply Sirona Endodontics, Ballaigues, Switzerland) using continuous clockwise rotation at 300 rpm and 2.5 Ncm. In all rotary systems, the final instruments used for canal preparation correspond to a tip size of 30.

All groups followed the same rising protocol using 5 mL disposable plastic syringes with 27-gauge needles that were close-ended and had rounded tips with side holes (Endo—Top, Cerkamed, Stalowa Wola, Poland). For each canal, after each instrument, 1 mL of 5.25% NaOCl (CHLORAXiD 5.25%, NaOCl, Cerkamed, Stalowa Wola, Poland) was applied, and as final irrigation, canals were flushed with 5 mL 17% ethylenediaminetetraacetic acid (EDTA), 2.5 mL physiological saline, 5 mL 5.25% NaOCl followed by a final rinse with 2.5 mL of physiological saline. The solutions were manually activated with the use of a gutta-percha (GP) cone reaching 1 mm shorter than the established WL.

In the case of multi-visit treatment, if necessary, calcium hydroxide (Calcipast, Cerkamed, Stalowa Wola, Poland) was used as an intracanal dressing. In the absence of symptoms of infection and pain, canals were rinsed with protocol described for a single visit appointment.

After drying canals with paper points, the final root canal obturation was performed with GP and AH plus (Dentsply Maillefer, Ballaigues, Switzerland) as a sealant using the cold lateral compaction technique. Next, the GP was cut off with a heated plugger, and the cavity was cleaned with isopropyl alcohol. The cavity was temporarily restored with a Fuji IX glass ionomer (GC, Tokyo, Japan) between the visits.

### 2.3. Assessment of the Root Canal Filling

**Figure 1:**
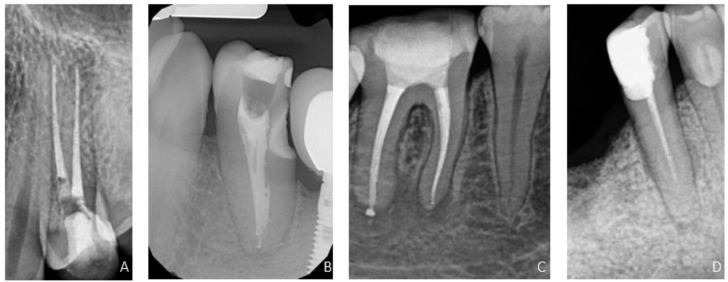
RVG images showing evaluation parameters. (**A**) Adequate length and density, (**B**) Inadequate density, (**C**) Overfilling, (**D**) Short-filling.

A retrospective randomized double-blind comparison study was conducted to assess the quality of the RCT. An X-ray with an X-ray positioning holder was performed before the final restoration of the tooth. X-ray images were taken with GENDEX Expert DC (KaVo, Biberach/Riss, Germany). The images were evaluated using the VixWin Platinum software (KaVo, Biberach/Riss, Germany). The distance between the end of the canal filling and the radiographic apex of the tooth root was measured. All data were coded and blindly assessed by two endodontists (K.P., M.R.). The two examining operators were calibrated before the examination. The calibration was performed on 30 cases. The quality of the filling on the X-ray was assessed according to ESE standards [9]. The assessment method of radiographs was a modified version of the technique introduced by Balto et al. [15]. The quality of root filling was evaluated according to two parameters: length and density (Table 1, Figure 1). A root canal filling was identified as acceptable when both parameters were satisfactory.

### 2.4. Statistical Analysis

All statistical analyses were performed with the statistical software package Statistica v. 13.1 (StatSoft, Inc., Tulsa, OK, USA). The normality test was performed using a Shapiro–Wilk test. The analysis of distribution of teeth and diagnoses between groups and differences in number of visits were conducted with use of a Kruskal–Wallis test. The statistical analysis of length and homogeneity of root canal filling was performed using the chi-squared test. The comparison between two groups (maxilla/mandible) without normal distribution and the gender and age of patients included in the study was analyzed with the Mann–Whitney U test. In all cases, statistical significance was considered at *p* < 0.05.

## 3. Results

### 3.1. Quality of Root Canal Filling

#### 3.1.1. Length

A total of 1733 obturated canals of 900 treated teeth were evaluated. The overfilling was observed most frequently in the palatal canal of first maxillary molars, while short-filling was observed in the mesio-buccal canal of first mandibular molars. Moreover, the material was extended beyond the apex more often in the case of diagnosis of periapical tissue inflammation. The number of adequate obturated canals was statistically significantly higher than the short- and overfilled (*p* < 0.05) (Table 2). The analysis of the discrepancy between the observed and expected numbers showed that in the case of endodontists, the material was overfilled more often than in the case of other groups. In the group of 4th year students, short-filling of the canals was observed significantly more often than in other groups (*p* < 0.05).

#### 3.1.2. Homogeneity

**Figure 2:**
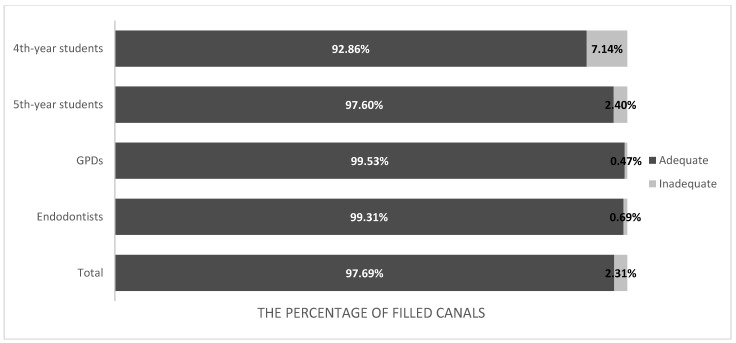
The distribution of density of canal filling in evaluated groups.

The analysis showed a statistically significant relationship between the inadequate filling of the canal in the studied groups (*p* < 0.0001). In the group of 4th year students, non-homogeneous filling of the treated canals was observed significantly more often than in other groups. The distribution of density in individual groups is shown in Figure 2.

Moreover, no statistical relationship was determined between the homogeneity and the length of the root canal filling in all evaluated groups.

### 3.2. Number of Visits

**Figure 3:**
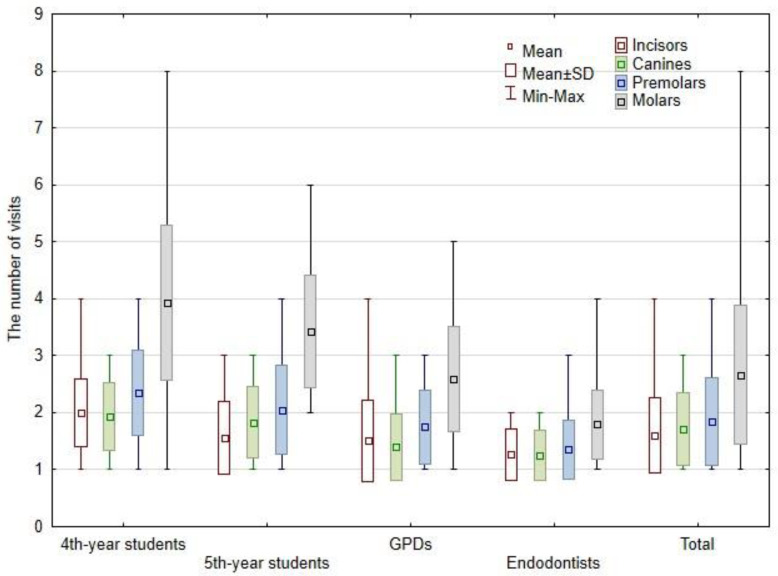
The mean value of number of visits in evaluated groups.

The mean value of visit is presented in Figure 3. The Mann–Whitney U test revealed that endodontists needed statistically significant fewer visits than other groups (*p* < 0.001). Moreover, GPDs needed statistically significant fewer visits than 4th year students (*p* < 0.001). The mean number of visits was significantly higher in the mandible (2.12 visits per tooth) than in the maxilla (2.01 visits per tooth) (*p* < 0.05). One-visit treatment was carried out in 12.04% of cases treated by 4th year students, in 26.15%—by 5th year students, in 32.06%—by GPDs, and in 51.36%—by endodontists.

The highest average number of visits for all teeth groups was recorded for 4th year students: 2.00 visits per incisor, 1.93 visits per canine, 2.34 visits per premolar, and 3.93 visits per molar. On the other hand, the lowest number of visits was noted for endodontists: 1.27 visits per incisor, 1.25 visits per canine, 1.35 visits per premolar, and 1.79 visits per molar. The treatment of incisors performed by 4th year students demanded statistically significantly the most visits when compared with other groups (*p* < 0.001). Next, the mean number of visits to treat canines by 4th year students was statistically greater in comparison to GPDs and endodontists (*p* < 0.05). In addition, the number of visits to treat canines was statistically larger for 5th year students when compared to endodontists (*p* < 0.05). However, the number of visits to treat premolars was significantly lower for endodontists than for the other study groups (*p* < 0.001). Moreover, the treatment of premolars by 4th year students demanded statistically larger numbers of visits than by GPDs (*p* < 0.01). Next, the mean number of visits to treat molars by students (4th and 5th year) was statistically greater in comparison to GPDs and endodontists (*p* < 0.001). The treatment of molars by GPDs demanded a statistically larger number of visits than by endodontists (*p* < 0.001). Moreover, no statistical relationship was determined between the number of visits and the homogeneity/the length of the root canal filling in all evaluated groups.

### 3.3. Type of Instruments

In total, 434 of the 900 teeth were prepared with hand files, and 466 were prepared using rotary instruments. The rotary systems were used only in the group of GPDs and endodontists. The distribution of rotary systems used is presented in Table 3. The ProTaper Next files were statistically more often used than other rotary instruments (*p* < 0.05).

### 3.4. Distribution of Teeth and Diagnoses

The age of the patients ranged from 6 to 86 years (mean amounted up to 47.61), with a gender distribution of 42.36% male and 57.64% female. Gender and age were not statistically significant (*p* > 0.05).

During the assessed period (2017–2019), a total of 900 primary RCTs were performed. The distribution of tooth groups and diagnoses in each group are presented in Table 4 and Table 5, respectively. No significant statistical differences were found between study groups regarding the distribution of teeth and diagnoses (*p* > 0.05).

## 4. Discussion

The present study compared the quality of the final root canal filling after primary root treatment performed by 4th and 5th year dentistry students, GPDs and endodontists. The quality of the final obturation on the X-ray was estimated according to the guidelines of the ESE [12]. The study evaluated length and density. Additionally, the number of visits, type of instruments used, and demographic data (distribution of teeth and diagnoses) were assessed.

### 4.1. Quality of Root Canal Filling

The quality assessment of the endodontic treatment carried out by dentistry students as well as GPDs and endodontists was evaluated in the literature [12,16,17,18,19,20,21,22,23,24,25,26,27,28,29,30,31,32,33,34,35,36,37,38,39,40,41].

Electronic and radiographic methods are recommended to determine the WL. According to the ESE, the WL should normally be confirmed with radiographs [9]. In the present study, the WL was confirmed both radiographically and with apex locators. The accuracy of modern apex locators measurement is undisputable [42,43]. Additionally, WL measurements with an apex locator showed higher accuracy than conventional periapical radiographs [43,44]. However, periapical lesions seemed to influence the measurements with apex locators [45]. According to another study, the precision of electronic measurement depends on the generation of apex locator and the type of irrigation used during RCT and is not affected by the status of the pulp tissue [46]. In the present study, the working length was confirmed using an apex locator with 5.25% NaOCl in root canal, similarly as in other studies [13,16].

The main criteria used in assessing the quality of RCT are the length and density of final obturation [9,10]. In the current study, the overall quality of RCT amounted up to 81.36%—for length and 97.69%—for density. In total, 14.60% and 4.04% of cases were overfilled and short-filled, respectively. In this investigation, not only the extrusion of gutta-percha cones but also any amount of sealer beyond apex was considered as overfilling. Various studies have investigated the radiographic quality of root canal fillings performed by clinical dental students [17,37,38,39,40,41]. In this research, the adequate length and density of RCT performed by 4th-year students were found in 81.71% and 92.86%, respectively. These results are in contrast with others showing higher [18,19] or lower percentages [20,39]. In the mentioned studies, the RCT of maxillary and mandibular teeth was performed [18,19,20,39], root canal shaping was carried out with the step-back technique [19,20] and root canal obturation was accomplished with the lateral condensation technique of gutta-percha [18,19,20,39] or with a single-cone technique [18]. In the current study, for 5th year students, the adequate dentistry and homogeneity were observed in 86.67% and 97.60%, respectively. Unlike in other research, the percentage of acceptable length and density performed by senior dental students was lower (59.48% vs. 50.76%) [21]. However, for incisors and molars, the proper length (81.71–86.67%) and density (92.86–97.60%) in the present and other studies were similar [22,23,24]. The observed discrepancies in outcomes may be only explained by the different types of endodontically treated teeth and the assistant–student ratio because endodontic procedures (canal preparation, MAF, WL measurement, rinsing solutions) were the same as in the present survey. Another study contradicts these results, where the lower percentage of adequate length (69%) and homogeneity (42.7%) was reported [34]. The lower results of that study [34] could be influenced by the type of treated teeth (mainly premolars and molars) and the combination of endodontic procedures with restorative treatment carried out in a multidisciplinary clinic (including prosthodontics). Therefore, all procedures performed during RCTs might not have been directly supervised by trained endodontists.

In the current study, the adequate length and density were noted in 85.75% and 99.53% for GPDs, respectively. The lower percentage (70.1%) of the adequate length of root canal filling was found for students of the postgraduate program in endodontics [16]. This difference could be associated with the different instrumentation and obturation techniques used in RCT in both studies. The inferior results of the mentioned survey could result from the lower number of treated molars in the present study (43.06% vs. 56.2%). In another research study where molars were treated in a group of GPDs, the adequate length and density were noted in 31.3% and 62.7%, respectively [27]. Interestingly, another study reported 90.3% cases with adequate obturation in the GDPs group [25]. However, in the present study, the results were similar to those reported by others (length—84.3% and dentistry—98%), where RCT was performed by postgraduate students [23,39]. These data are in contrast with the results reported in a retrospective study assessing the obturation quality of primary RCTs of molar teeth performed by GPD, where an acceptable quality of root filling was only found in 31.3% [27]. In contrast, in Saudi Arabia, GPDs performed RCT with adequate length and density in 46.4% and 75.8%, respectively.

In this investigation, the adequate length for endodontists was noted in 74.48%, which is in agreement with other research [26], but it is in contrast with others showing higher (86–93.55%) [28,29] or lower percentages (41.2–62.5%) [27,47]. Similarly to these results, the adequate quality of root fillings (length and density) performed by Australian endodontists was found in 77.4–91.0% [30]. These discrepancies can be related to different NiTi rotary systems, obturation methods (lateral condensation of cold gutta-percha vs. single cone technique) and number of operators who performed RCT (one vs. more). In the present study, the short-filling of root canals among endodontists was reported in 3.28% of cases, similarly to others [26,28]. However, it is in contrast with the studies where mainly posterior teeth were treated (short-filling in 17.7–18.8%) [27,47]. The short-filling may occur due to the anatomical complexity of mandibular molars, underestimation of working length, inconsistent reading of the apex locator in a canal with lateral canals and deltas, instrumentation mishaps (ledges, blocking, failure to maintain apical patency), inadequate chemo-mechanical preparation and an incorrectly matched master apical cone [27]. In this study, overfilled canals were found in 22.2%, similarly to other research [26,47]. More favorable results (overfilled canals in 6.45%) were found in the study where only one specialist performed the RCT (2000 RCTs) [29]. However, the lower percentage of overfilled canals was reported by others (4.4%) [27]. Further analysis of teeth groups in the above-mentioned study [27] indicated that overfilling was observed more often in maxillary molar teeth, whereas short-filling was observed in mandibular molar teeth, which is supported by the current study. The overfilling can be observed due to the overestimation of working length, the apex locator may give false results in roots with apical resorption, overpreparing of the canal, incorrectly matched master apical cones, and excessive pressure on the spreader during the lateral condensation technique, especially after rotary instrumentation [27]. Additionally, the final obturation of the root canals beyond the apex may occur more often in the case of periapical tissue inflammation [48]. It should be emphasized that the small extrusion of a sealer is generally well-tolerated by the periapical tissues [49,50,51]. However, some authors found a higher risk of non-healing lesions in cases with sealer extrusion [52,53].

According to the literature, inadequate root canal filling with voids was the most affected by bacterial leakage [1]. Residual microorganisms inducing the root canal infection were associated with apical periodontitis [54]. In the present study, the adequate density for endodontists was found in 99.31%. However, poorer results were reported in previous studies (49.6–81.3%) [27,28,47]. Interestingly, 97% of cases with adequate obturation (no voids and proper length) were treated by endodontists [25], while in this study, a lower percentage was (76.26%) observed. This might be related to the different generation of NiTi rotary systems used, where all operators (six endodontists) used rotary NiTi instruments [30] and had different experience (4–26 years). However, the retrospective study assessing the obturation quality of primary RCTs of molar teeth performed by endodontists reported an acceptable quality of root filling in 50.4% [27]. These unfavorable results might be attributed to the filling method (single cone technique) and subsequent misfit between cone and master file size and taper causing voids [27].

### 4.2. Number of Visits

In the current study, the 4th and 5th year students conducted multi-visit treatments in 87.96% and 73.85%, respectively. Comparable results of multi-visit treatments (86.3%) carried out by the undergraduate students were reported [55]. However, another study noted a 1:1 ratio between single-visit and multi-visit treatment performed by undergraduate students [20]. In the present study, the treatment of lower teeth demanded more visits than that of upper teeth. This might be attributable to the anatomical difficulties and difficulties related with an ineffective inferior alveolar nerve block of mandibular molars with pulpitis.

The multi-visit treatment was also mainly performed by GDPs (67.94%), which was in contrast with other studies (92.2–96.2%) [32,56]. In the present study, the one-visit treatment was carried out by endodontists in 51.36%. However, a significantly lower percentage was reported in the literature (4.7–37.15%) [29,30,32,56]. In the present study, most of the one-visit treatment was performed by endodontists, while other operators preferred the multi-visit treatment. The rotary systems used in the group of GPDs and endodontists might have shortened the number of visits in this study.

The issue of one- and multi-visit endodontic treatment has been under dispute for a long time. There is still no consensus regarding this matter [57,58,59]. However, with the introduction of new heat-treated files and techniques, it seemed that the single-visit procedure has become a good treatment option [58,60,61]. According to the literature, both procedures had similar success rates of RCT regardless of the diagnosis (pulp vs. periapical tissue) [61,62,63]. During multiple-visit RCT, usually, an antiseptic medication (calcium hydroxide) is placed in the root canal system for further disinfection of the canals between treatment appointments [64,65]. In contrast, in single-visit RCT, the root canal system is obturated directly after shaping and cleaning without any antibacterial medication. The higher concentration (5.25%) of NaOCl presents faster and greater capacity in dissolving the organic matter, lubrication, and bactericidal effect. In addition, the number of visits might also depend on the dexterity and skills of the operator [32,66]. It is a well-known the fact that experience and specialty training strongly influenced the decision making during RCT provided by GDPs and endodontists [67,68]; the data also showed that root morphology was significant [69]. Other common reasons why GDPs and endodontists choose multiple-visit treatment were the healing effect of inter-appointment medications, lessening the symptoms of apical periodontitis and the shorter duration of the appointment [32].

### 4.3. Type of Instruments

According to ESE general guidelines [9], the root canal after preparation should be tapered, microorganisms should be eliminated, and debris should be removed [2]. The crucial goal of RCT is the reduction in intracanal infection. It could be accomplished by means of a proper chemo-mechanical preparation with irrigation, hand files or rotary systems [2,70]. The rotary canal instrumentation exhibited a similar clinical and radiographic success rate, diminished procedural errors, more predictable canal shape and an increased pace of work when compared to the manual instrumentation technique [71,72,73].

It was suggested that the RCT performed by undergraduate students as unexperienced operators should be performed only in cases with minimal complexity at the beginning of clinical practice [13,27,74,75,76]. Similarly, in the present study, students performed manual instrumentation with the step back technique [40,74,77,78]. In contrast, according to other studies, undergraduate students utilized NiTi rotary instruments and the crown-down technique [12,13,33,35].

In the present study, 48.22% of endodontically treated teeth were prepared with hand files, while 51.78% were prepared with rotary instruments. The rotary systems were used only by GPDs and endodontists. However, in a previous study [16], postgraduate students of program in endodontics performed RCT with manual and rotary instrumentation in 20.2% and 79.8%, respectively. The technique used by the endodontists and the GDPs for chemo-mechanical preparation in the current study was the crown-down or single length technique, which is in accordance with other research [27,28,79]. However, another study reported that all canals were prepared with stainless steel files and the step-back technique [29]. However, the current literature indicates that endodontists conducted only difficult cases of RCT using rotary instruments [28,30,80].

### 4.4. Distribution of Teeth

In the present study, the 4th year students treated more often incisors (29.63%) and premolars (29.17%), while the 5th year students treated more often incisors (30.73%) and molars (30.28%). A very similar distribution was presented by other scientists [24,36,81]. According to other studies, two-thirds of treated teeth by 4th and 5th year students were premolars and molars [23,37], while one study presented a uniform distribution of teeth treated by students [38]. According to the ESE undergraduate curriculum guidelines for endodontology [10], students should gain adequate experience in the treatment of anterior, premolar, and uncomplicated molar teeth. The quality of RCT performed by students depended on the number of endodontic procedures performed during pre- and clinical training, the treatment protocol used, and the assistant–student ratio [10,12,13]. Moreover, the teaching staff should be specialists or have a special interest in endodontology [10]. It was claimed that dentistry students should perform only RCT with minimal complexity [13,27,74,75,76]. However, upon graduation, dentists should demonstrate both in-depth theoretical knowledge and appropriate clinical skills acquired during preclinical and clinical classes in the field of endodontics.

In this study, premolars and molars were the most frequently treated teeth by GDPs and endodontists; similar data were presented by previous authors [30,36,47,80,82]. However, other researchers indicated that GDP more often conducted RCT of anterior teeth [77], and endodontists treated only posterior teeth [25]. Moreover, simple cases of RCT were more often performed by GDP than by endodontists [31,82,83,84]. Interestingly, another study claimed that anterior teeth were treated endodontically by a specialist in 52.1% [30]. According the ESE report, a dental practitioner is expected to treat effectively pulpal and periapical diseases and have a basic knowledge of endodontology [10]. In contrast, a specialist should possess highly developed technical and clinical skills to perform complex primary root canal treatment, re-treatment or endodontic surgery [14].

### 4.5. Distribution of Diagnoses

The most common diagnosis prior to primary RCT in the current study was pulpitis (55.78%), less often was periapical tissue disease (43.67%) and the least frequent was endo-perio lesion (0.56%). These data coincided with the available literature [29,38,39]. Various studies reported that primary RCT was performed by postgraduate students of the program in endodontics, and additionally, pulpitis was diagnosed more often than non-vital pulp [16,80]. However, other studies did not support these findings [38,78]. Interestingly, it was claimed that one-third of endodontically treated teeth were diagnosed as irreversible pulpitis, while half were diagnosed as pulp necrosis associated with periapical radiolucency [38,40], and endodontic–periodontic lesions were diagnosed rarely (2%) [38]. According to the undergraduate curriculum guidelines for endodontology [10], GDPs should be familiar with the management of pulp and periradicular disease and be able to perform RCT of uncomplicated anterior and posterior teeth. An endodontist is a clinically competent practitioner who performs primary and secondary RCT of teeth with complicated anatomy, infected root canal systems, and periapical infection, with the use of magnification (dental operating microscope) [35] and modern endodontic tools and devices.

Moreover, the limitations of the present study should be acknowledged. The distribution of teeth in the study groups should be more consistent. Premolars and molars were the most frequently treated teeth by GDP and endodontists; meanwhile, anterior teeth were the most frequently treated teeth by the undergraduate students. Another limitation is the skill of the operator and the analysis of the RCT quality in two-dimensional images. Conventional radiographs compress three-dimensional anatomical structures into a two-dimensional image, greatly limiting diagnostic performance. Additionally, root canals are visualized in the mesio-distal plane only, and the bucco-lingual plane may not be completely appreciated [85]. Furthermore, anatomical noise, geometrical malformation, and two-dimensional imaging may cause impaired diagnosis [86]. Another limitation of the present study is the differentiated instrumentation technique. RCT performed by undergraduate students was conducted with manual instrumentation, while by GPDs and endodontists, it was conducted with rotary instrumentation techniques. Moreover, further studies comparing other evaluation methods, instruments, canal shaping and obturation techniques should be carried out. Next, corresponding research, investigating the quality of endodontic retreatment, and respective comparisons should be performed.

## 5. Conclusions

Within limitations of the study, the following can be stated:

1. The larger number of visits and the lower quality of treatment was observed for 4th-year students than for other groups; in contrast, endodontists needed the lowest number of visits to complete RCT and more often overfilled teeth than the other operator groups.
2. Interestingly, no difference in quality (homogeneity and length) of root canal filling between 5th-year students, GPDs, and endodontics was noted.
3. Endodontists and GDPs more often performed RCT of teeth with more complicated anatomy (premolars and molars).
4. The overfilling was observed most frequently in the palatal canal of first maxillary molars and in the case of periapical tissue inflammation, while short-filling was observed most frequently in the mesio-buccal canal of first mandibular molars.
5. The treatment of lower teeth demanded more visits than that of upper teeth.

## Figures and Tables

**Table 1:** Evaluation of parameters of root canal filling.

Parameter of Root Canal Filling	Criteria	Definition
Length	Adequate	Root filling ≤2 mm from radiographic apex
	Overfilling	Root filling beyond the radiographic apex (gutta-percha cones or/and sealer)
	Short-filling	Root filling >2 mm from radiographic apex
Density	Adequate	Voids absent, homogeneous root filling
	Inadequate	Voids present, heterogeneous root filling

**Table 2:** The distribution of root canal filling length in each group.

	Number of Root Canals			
Group/Diagnoses	Adequate	Overfilling	Short-Filling	Total
4th year students	286 (81.71%)	40 (11.4%)	24 (6.86%)	350
5th year students	325 (86.67%)	41 (10.9%)	9 (2.40%)	375
GPDs	367 (85.75%)	43 (10.0%)	18 (4.21%)	428
Endodontists	432 (74.48%)	129 (22.2%)	19 (3.28%)	580
**Total**	1410 (81.36%)	253 (14.60%)	70 (4.04%)	1733

**Table 3:** The rotary systems used during RCT.

Group/Rotary System	ProTaper Next (Dentsply Maillefer)	Mtwo (VDW)	E3 Azure (Poldent)	DC-Taper 2H (SS White)
	Number of Teeth			
GPDs	184	6	1	18
Endodontists	208	48	1	0

**Table 4:** The distribution of teeth in each group.

Group/Teeth	Incisors	Canines	Premolars	Molars	Total
4th year students	64 (29.63%)	43 (19.91%)	63 (29.17%)	46 (21.30%)	216
5th year students	67 (30.73%)	41 (18.81%)	44 (20.18%)	66 (30.28%)	218
GPDs	41 (19.62%)	25 (11.96%)	53 (25.36%)	90 (43.06%)	209
Endodontists	52 (20.23%)	16 (6.23%)	73 (28.40%)	116 (45.14%)	257
**Total**	224 (24.89%)	125 (13.89%)	233 (25.89%)	318 (35.33%)	900

**Table 5:** The distribution of diagnoses in each group.

Group/Diagnoses	Pulp	Periapical	Endo-Perio	Total
	Number of teeth			
4th year students	133 (61.57%)	83 (38.43%)	0	216
5th year students	113 (51.83%)	105 (48.17%)	0	218
GPDs	107 (51.20%)	97 (46.41%)	5 (2.39%)	209
Endodontists	149 (57.98%)	108 (42.02%)	0	257
**Total**	502 (55.78%)	393 (43.67%)	5 (0.56%)	900

## Data Availability

Not applicable.
